# Impact of COVID-19 on Children and Young Adults With Type 2 Diabetes: A Narrative Review With Emphasis on the Potential of Intermittent Fasting as a Preventive Strategy

**DOI:** 10.3389/fnut.2021.756413

**Published:** 2021-10-28

**Authors:** Hala K. Elmajnoun, MoezAlIslam E. Faris, Suma Uday, Shaun Gorman, James E. Greening, Parvez I. Haris, Abu-Bakr Abu-Median

**Affiliations:** ^1^Leicester School of Allied Health Sciences, Faculty of Health and Life Sciences, De Montfort University, Leicester, United Kingdom; ^2^Department of Histology and Medical Genetics, Tripoli University, Tripoli, Libya; ^3^Department of Clinical Nutrition and Dietetics, College of Health Sciences, Sharjah Institute for Medical and Health Sciences (RIMHS), University of Sharjah, Sharjah, United Arab Emirates; ^4^Department of Endocrinology and Diabetes, Birmingham Women's and Children's Hospital, Birmingham, United Kingdom; ^5^Department of Paediatrics, St Luke's Hospital, Bradford, United Kingdom; ^6^University Hospitals of Leicester NHS Trust, Leicester Royal Infirmary, Leicester, United Kingdom

**Keywords:** type 2 diabetes, children, young adults, COVID-19, Ramadan fasting, intermittent fasting

## Abstract

**Background:** The world is still struggling to control the COVID-19 pandemic caused by the severe acute respiratory syndrome coronavirus 2 (SARS-CoV-2). The level of uncertainty regarding the virus is still significantly high. The virus behaves differently in children and young adults. Most children and adolescents are either asymptomatic or have mild symptoms. They generally have a very good prognosis. However, it is not well-known whether children and young adults with type 2 diabetes are at risk of getting a severe infection of COVID-19. Many Muslim children with type 2 diabetes have been performing dawn to dusk fasting during the month of Ramadan, before and during the COVID-19 pandemic, and the impact of this on their health has not been well investigated. Previous studies in adults have suggested that intermittent fasting may be beneficial in different ways including reversal of type 2 diabetes and prevention of COVID-19 infection.

**Objective:** The primary aim of this narrative review is to summarise the impacts of the COVID-19 pandemic on children and young adults with type 2 diabetes, and to identify the knowledge gaps in the literature. It also explores the potential of intermittent fasting in reversing the pathogenesis of diabetes and highlighting how this approach could prevent these patients from developing chronic complications.

**Methods:** This narrative review has been produced by examining several databases, including Google Scholar, Research Gate, PubMed, Cochrane Library, MEDLINE (EBSCO), and Web of Science. The most common search terms used were “COVID-19 AND Children”, “SARS-CoV-2 AND/OR Children”, “COVID-19 AND Diabetes” “COVID-19 Epidemiology”, “COVID-19 AND Ramadan fasting”, “COVID-19 and Intermittent fasting.” All the resources used are either peer-reviewed articles/reports and/or official websites of various media, governmental and educational organisations.

**Results:** Having reviewed the currently limited evidence, it has been found that the incidence of COVID-19 among children with type 2 diabetes seems to be not much different from children without diabetes. However, these patients are still vulnerable to any infection. Several studies have reported that prevention programmes such as intermittent fasting are effective to protect these groups of patients from developing any complications. Moreover, observing Ramadan fasting as a type of intermittent fasting could be beneficial for some children with established diabetes, prediabetes and people at risk.

**Conclusion:** Children and young adults with type 2 diabetes are not at risk of severe COVID-19 infection as the case in adults with diabetes. More research is needed to identify the impact of COVID-19 and to investigate the efficacy and safety of intermittent fasting, including Ramadan fasting, among these age groups. Implementing these cost-effective programmes may have a great impact in minimising the incidence of diabetes. Moreover, this could be effective particularly at prediabetes stage by preventing these people from going onto develop type 2 diabetes and taking medications for the rest of their life and protecting people from complications linked to disease and infection.

## Introduction

The global potential impacts of the coronavirus disease 2019 (COVID-19) caused by SARS-CoV-2 on children and young adults have been examined. It has been reported that the disease is less prevalent among these age groups, about 1–2% of the total cases (1, 2). They seem to have less risk of catching the infection and there is a very low mortality rate in comparison to adult people (3–5). In contrast, Dong et al. (4) have concluded that children and young adults are similar to adults in terms of their sensitivity or their risk to COVID-19 infection, however, the course of the disease is unusual. Typically, for children and young adults, the disease is mild and less severe compared to adults and infants (less than a year) and they often recover within 1–2 weeks (5). Moreover, it has been noticed that most children confirmed as having COVID-19 are asymptomatic (5). However, severe to moderate symptoms have been recorded among infants who are sensitive to the infection (4). Moreover, new onset of type 1 diabetes (T1D) related to COVID-19 among children have been reported in the UK and in the US (6, 7). However, this has not been noticed yet among children with type 2 diabetes (T2D).

Understanding the impacts of the COVID-19 infection on children and young adults with T2D is one of the aims of this narrative review article. It is widely assumed that these patients are at the same risk as their peers who do not have T2D (8). Even though pathological alterations develop in these patients, which suggest that they might be at risk of getting severe COVID-19, no evidence has been provided to support this theory. These particular patients have not been recognised as a high-risk group for developing severe COVID-19, which is opposite to the case among adults with T2D (8, 9). It is not well known yet why the disease is mild among children, however, there have been some theories to explain this (10). Moreover, there is a great deal of debate about whether asymptomatic children can transmit the infection to adults and other children (with or without health problems), and for how long the asymptomatic children could be considered as a source for transmission of the infection (11, 12). Statistically, T2D among children and young adults has significantly increased in recent years (13–15). The COVID-19 pandemic and its associated circumstances (recurrent lockdown measures and movement restrictions) could have a substantial impact on increasing the percentage of these patients in the world. Taken together, there remain some open questions about whether these patients are at risk or not and how these patients could be protected to prevent them from developing any complications due to the current COVID-19 pandemic.

The level of uncertainty regarding this pandemic is significantly high. Changing dietary and lifestyle behaviours, such as physical exercise, a healthy diet, and the practice of intermittent fasting (IF) may play a role in boosting immunity (16). Encouraging all the habits that help to boost immunity could improve the disease prognosis in general. One of these practices is Ramadan intermittent fasting (RIF) and other types of IF (17). This review will highlight the importance of implementing these strategies. The beneficial role of RIF and other types of IF in fighting infections and boosting immunity has been reported elsewhere (18–20). Moreover, Hannan et al. (21) have recently reviewed the importance of IF and how it could be used as a potentially protective approach to fight COVID-19. Furthermore, Faris et al. (22) indicated that RIF positively affects the body's immunity by changing different related elements, including oxidative stress and inflammation, metabolism, body weight, and body composition. Thus, this review will discuss and evaluate the current literature related to the effects of Ramadan fasting (RF) on human health and patients with T2D and how this could be applied during the current pandemic.

Several studies have shown that RF was associated with a positive impact in controlling blood glucose and weight loss among patients with T2D in adults (23–25). However, the findings of other studies suggested that RF could increase the risk of hypoglycaemia in some of these patients, while could not in others (26–28). This variation could be ascribed to many factors such as season of Ramadan month, fasting time duration, pre-fasting education, geographical location and the duration of time since diagnosis with the disease (29). Thus, this review has hypothesised that some children who are eligible to fast according to Islamic regulations on RF, which usually starts around 12 years old or reaching puberty, will benefit from RF and the effects could be the same as in adults. This is based on the fact that the pathogenesis of T2D in children is similar to that of adult patients (30). On the other hand, some may suffer due to the severity of their medical condition, poor diet, lack of activity, and anxiety. In addition, this narrative review has suggested several precautions could be taken before the month of Ramadan, such as intensive education programmes, adjusting medication, physical exercise, and avoiding missing follow-up appointments with the medical care professionals. This has to be implemented with clear communication from health care providers. To test this hypothesis, it is necessary to discuss the current scientific evidence on the risk of COVID-19 among children and young adults amongst patients with T2D compared to healthy people. Besides, the effects of RF and its long-term effects among these age groups of the population will be examined.

## Methods

This narrative review has been produced by examining several databases, including Google Scholar, Research Gate, PubMed, Cochrane Library, MEDLINE (EBSCO), and Web of Science. The most common search terms used were “COVID-19 AND Children”, “SARS-CoV-2 AND/OR Children”, “COVID-19 AND Diabetes” “COVID-19 Epidemiology”, “COVID-19 AND Ramadan fasting”, “COVID-19 and Intermittent fasting.” All the resources used are either peer-reviewed articles/reports and/or official websites of various media, governmental and educational organisations.

## Epidemiology Of Covid-19 Among Children and Young Adults

Based on the epidemiological summary, which has been published and is updated regularly by the Royal College of Paediatrics and Child Health, children and young adults can be affected by the COVID infection, however, the number is very small (≤ 5%) in comparison to adults and adolescents who are more susceptible to the disease than younger children (31). Another UK study reported that children (less than 16 years old) testing positive for COVID-19, represented a very low percentage (1.1%) among over 35,000 children tested (32). This study was conducted between January and May 2020. Moreover, a retrospective study in Italy has reported that children who had COVID-19 were only 1% of the total cases at the beginning of the pandemic and that no deaths have been recorded among this age group (33). Similarly, a multicentre cohort study, involving 25 countries in Europe, has reported that the mortality rate was very small-−0.36% (4/582)—among children and teenagers with COVID-19 (34) including children with chronic medical problems. Generally, the course of the disease is mild, and very few numbers had moderate to severe symptoms. Moreover, the risk of mortality is extremely rare (0.01–0.1%), which is quite similar to the incidence of deaths due to seasonal flu per year (31). A systematic review was conducted worldwide during May 2020 and reported that children and young adults (up to 21 years old) with COVID-19 had a very good prognosis and most of the cases recovered completely, including people with pre-existing medical problems (35). They found that the mortality rate was just 0.09% among a total number of around 8,000 confirmed cases. This result was based on analysed data from healthy children and children with comorbidities (35). It seems that the younger the age, the better the outcome if someone has COVID-19.

According to Diabetes UK, children with diabetes can become infected with COVID-19 virus, however, the risk of developing severe illnesses is extremely rare (36). Nevertheless, these children and adolescents with diabetes are still vulnerable to the COVID-19 infection and careful precautions should be in place and close health care observations are highly recommended for these patients. This is particularly so in patients with uncontrolled blood glucose and who have a secondary complication of diabetes. Even though the COVID-19-related mortality rate has increased sharply among adults with diabetes, the risk of death in children with diabetes has not been recorded yet in the UK (36, 37). Furthermore, it has been reported that most of the hospital-admitted children (with comorbidities) who were confirmed to have COVID-19, were from ethnic minority groups, including Asian, and Black and other minor ethnicities (38), indicating that ethnicity could be considered as an independent risk factor for making the disease hard to control. Authors have suggested that this might be greatly influenced by the cultural and behavioural differences among these societies (38). Recently, it has been reported that many of the children and adolescents (less than 19 years old) who had developed paediatric multisystem inflammatory syndrome in children, were not of white ethnicity at 64% (39). This has also been proven by the multicentre prospective cohort study in the UK where around 651 patients with acute COVID-19 were admitted to the emergency departments. Only six died in the hospital, which is only 1 % of the total number and all of them had previous chronic illnesses (39).

It is known that children from different communities are not tested as frequently as adults. Therefore, it is expected that more children are affected by the SARS-CoV-2 virus in all societies. This has been clearly seen by the significant surge in the numbers of affected cases amongst pupils and staff members in the second week of returning to schools in the UK (40). Consequently, this had an impact on the sharp increase in the number of COVID-19 cases in the whole country (41). Thus, it has been noticed that the available data do not reflect the true picture of COVID-19 in children and young adults (42). Furthermore, at the early period of the outbreak, COVID-19 tests were restricted and were mainly for children with severe symptoms and who required hospital admission.

More recently, it has been observed that the incidence of COVID-19 has increased significantly and steadily among young adults (10–29 years old) in the UK (43). It has been suggested that this could be related to the fact that the young adults are not following COVID-19 protection rules in terms of wearing masks and maintaining the recommended social distance; there is no evidence to support this explanation though (43). In a month, the number of cases of those in their teens increased by four-fold and it has risen around three times among people in their twenties (43). However, it has not been established whether these identified cases are all healthy individuals or whether they have chronic diseases such as T2D. Therefore, epidemiologically, the accurate number of infected children either healthy or patients with T2D is not well known in most countries. For example, locally, how many children are affected at a school in the UK, how many teachers are affected by COVID-19 at a school in the UK, and how many children with diabetes had COVID-19 during the whole pandemic? Researchers and the general public have been struggling to find the answers to all of these questions. Apparently, governments around the world are experiencing great challenges in terms of collecting accurate data and classifying these data by age and sex. Moreover, there still remains a substantial deficit in capacity to test for COVID-19 and availability of the more accurate PCR testing. Identifying accurate statistics is essential to apply the right prevention, management, and control strategies to overcome this pandemic.

## Children Who Have Been Confirmed as Having the Covid-19 Infection Either Mild or Asymptomatic - Why?

There is great uncertainty regarding the effects of COVID-19 on children and young adults. The risk of the disease has not been recognised even in patients with chronic diseases such as diabetes. It could be argued that the biological, immunological, and physiological mechanisms in children could play a key role in how children's bodies are behaving with—and responding to—the virus as this might be determined and modulated by the developmental phases of the endocrine, muscle and nervous systems (44). Lingappan et al. (10) reviewed varied scientific pieces of evidence, which indicated that children have a significantly lower expression of the *Angiotensin-converting enzyme 2* (ACE2) receptors, which are required for SARS-CoV-2 binding to the cells. Besides, they found that the level of expression of these receptors is directly correlated with age. Moreover, it has been reported that the virus is competing with other viruses in children's airway mucosa, which is preventing the entry of the virus (45).

Another theory that has explained why children have mild COVID-19, is the maturity of the immune system in adults compared to children and adolescents (46). The innate immune system is weaker among children and this is further associated with the lower activity of the immune cells such as macrophages, dendritic cells, and neutrophils (10). These cells are involved in the proinflammatory state and trigger several cytokines among adults with COVID-19, which in turn indirectly damage the lung tissue (10). It has been suggested that this immune overreaction is subtle or does not develop in children and young adults. Supporting this hypothesis, a study investigated the pathogenesis of SARS-CoV-2 using a mouse model to explore the difference in the immune responses between adult and young mice (47). They noticed that the virus induced severe inflammatory reactions only in adult mice and this was associated with serious respiratory complications including alveolar damage and pulmonary oedema. This could be the same case in SARS-CoV-2, however more research-driven data are needed to confirm this.

Moreover, children could be protected by the trained immunity that had developed due to some vaccines such as the bacillus Calmette-Guérin (BCG) vaccine (48, 49). Several previous researchers have reported that the BCG vaccination was associated with a significant decline in the incidence of respiratory tract infections and decreased the infant mortality rate [reviewed by O'Neill and Netea (49) and Pandit et al. (50)]. They showed that children could possibly have a powerful innate immune system as they are used to having recurrent viral infections. Consequently, the level of immunoglobulins is expected to be high and it is protecting them from getting the infection and developing severe illnesses (51). Also, it has been reported that the severity of pneumonia in children was significantly connected to the immune response (47). Cases of mild pneumonia in children were associated with the activation of CD8+ T cells and the adaptive immune response of the IL-10 (52, 53). Thus, understanding the mechanisms/reasons behind the mildness of the disease among children will pave the way for developing the means of tackling the disease and in creating preventive approaches against COVID-19, which could be applied among children, adults and people with chronic disorders (54).

All the above hypotheses could be applied to children with diabetes as well. However, these patients are still at risk of developing severe proinflammatory complications due to COVID-19 and on top of this most children with T2D are associated with obesity (55). Furthermore, high levels of proinflammatory cytokines in obese children have been reported such as IL-6 and IL-15 (56). This in fact could worsen the disease prognosis among these patients by increasing the risk of cytokines damaging surge. Therefore, theoretically, there is still a concern regarding children with obesity who have T2D diabetes, even though, currently this has not been recognised as is the case in adult patients. Furthermore, at the early stages of the pandemic, cytokine storm has been reported in eight critically ill children (ranged from 5 months to 15 years old) with no previous chronic diseases (57). Most of these children had direct contact with COVID-19-infected cases. Furthermore, Cho et al. (58) have shown that the dysregulation of some cytokines [resistin and plasminogen activator inhibitor 1 (PAI-1)] was associated with developing a new-onset of T2D among adults with prediabetes. However, this has not been identified in children and young adults yet. Therefore, precaution and well-controlled diabetes are inevitable among this group of population. In addition, several protective and preventive strategies to reverse T2D could be applied, such as introducing healthy diet programmes, practising IF, and encouraging physical activities. These will be discussed below in more detail.

## The Risk of Covid-19 Transmission From and on Children

The risk of COVID-19 infection transmission from children to adults has been a significant concern for many people and researchers. Moreover, much of the research up to now has been descriptive in nature. Wongsawat et al. (59) have investigated the risk of spreading the infection from children with COVID-19 to their household/carers. They concluded that there was no risk of the transmission of the COVID-19 from children (4 and 8 years old) to adult carers. However, this study was designed as a case series in which the number of cases was very limited, and the cases had mild symptoms (mild cold and with no fever) (59). On the other hand, another study in China has shown that children (mean age was 6 years) with non-severe symptoms of COVID-19 were associated with a risk of transmission to their parents, even though the risk was only 1% of the total studied cases (60). This was defined as ‘intrafamily transmission' (60). Besides, they noticed that about 50% of patients had SARS-CoV-2 RNA identified in their stool samples within 1 month of the start of the illness (60). Therefore, the authors have raised the warning that children could be a source of infection to others, adults and children, even after the symptoms have completely resolved. This could be related to the fact that the incubation period of COVID-19 infection among children is slightly longer than in adults (60, 61). Recently, evidence has reported that children are infectious to others even if they are asymptomatic or having mild symptoms (62, 63).

Thus, in terms of preventing the public transmission of this current pandemic, more investigations are vital. Furthermore, most of the infected children were secondary cases as a result of being exposed to adult cases (households) or travel-associated (60, 64). Therefore, it seems that children could be involved either way in human-to-human transmission and this will have an important role in Infection-Prevention-Control strategies for this pandemic. In a retrospective study using data from three hospitals in China, Qiu et al. (65) reported that 36 patients, under 16 years old, were confirmed to have COVID-19 within 2 months. The sources of infection for most of these cases (approximately 90 %) were from household contacts (65). Also, most of the patients in this study were admitted with moderate to mild symptoms and around 30 % were asymptomatic (65). Importantly, this highlights the point that a substantial number of asymptomatic children are hard to identify among communities as they lack the typical clinical and epidemiological features to tackle the disease transmission. Consequently, this feature could seriously increase the risk of making COVID-19 one of the community-acquired infections (57, 65). However, the ability of asymptomatic cases to transmit the infection to others remains unclear and further investigation is needed.

It has been reported that a considerable number of children with confirmed COVID-19 had typical radiographic features during the first few days of the infection or since they had been in contact with an infected person or a household (60). For this reason, all children who are asymptomatic and/or have mild symptoms and have a history of contact with infected people should be followed closely by their carers (parents and health care providers). However, such an approach might be hard to apply in some countries. Therefore, all these findings could have a negative impact on patients with chronic illnesses such as children and adolescents with T2D.

## Diabetes Epidemiology

All over the world, the incidence of diabetes has increased tremendously throughout the last decade. According to the International Diabetes Federation (IDF), it has been estimated that the number of patients with several types of diabetes, aged between 18 and 99 years, reached 451 million in 2017, and in 2045 this figure is projected to expand to 693 million worldwide (66). Furthermore, they estimated that there are around 352 million people worldwide who are pre-diabetic (who have impaired glucose tolerance) and this number is predicted to grow up to 531.6 million by 2045. These figures give an estimate that nearly half of all populations are either at prediabetes stage or undiagnosed cases and about 5 million deaths among the same age groups were due to diabetes during 2017 (66, 67). Globally, it has been predicted that 90% of patients who are diagnosed with diabetes have type 2 diabetes (68–70). Moreover, based on the last report that was published by the World Health Organization (WHO), the global number of diabetes (T1D and T2D) among young adults and adults, ≥ 18 years old, in 1980 stood at 4.7% and had remarkably grown to 8.5% by 2014 (71). This rise was associated with the increased incidence of numerous risk factors such as obesity and a sedentary lifestyle. Additionally, it was reported that in 2016, diabetes was the seventh cause of death in the world (71). Therefore, these warning statistics are expected to get worse during the current COVID-19 pandemic with the consequences of the recurrent lockdown measures.

According to the National Paediatric Diabetes Audit (2018–2019), it has been reported that the recent update for the prevalence of patients with T2D among children and young adults (<25 years old) in the UK was 790 (72). They indicated that this number was based only on the patients who were under the Paediatric Diabetes Units (PDUs) and did not include the patients who had been followed by primary care and private clinics. Besides, it was most predominant among girls whose ethnicities are non-white (72). Moreover, according to Diabetes UK, it has been reported that ‘there are more than 7,000 children and young adults under 25 with T2D in England and Wales' (73). Therefore, all these statistical findings confirm the issue that the number of children with T2D has substantially increased in comparison to other types of diabetes during recent years. It could be argued that compared to the total population in the UK, which is around 66 million, the incidence of T2D would be expected to be much higher than this figure (74). In addition to the current COVID-19 pandemic, the number of cases with diabetes and prediabetes among this age group is anticipated to be doubled by the end of the year. However, no recent statistics have been announced yet. Another important point to mention is that T2D at a younger age is associated with significant risks of vascular morbidity, recurrent fracture, and high mortality rate (75, 76). Therefore, highlighting these statistics is extremely important to provide valuable evidence to create new government policies/guides in agreement with the health care professionals. For instance, IF could be recommended for children who are at prediabetes stage as it has been recommended for adults (77). However, more research is needed in order to apply this to the medical practice. In addition, providing the optimal health care to this group of the population (during the current pandemic) should be seen as an urgent matter. For example, providing/sponsoring free virtual education events for parents and children in different societies would be beneficial. This could importantly prevent or minimise the epidemic rise of T2D.

## Effects of the Covid-19 Pandemic on Patients With Type 2 Diabetes Among Children and Young Adults

It has been reported that the risk of death and comorbidity progression is at the same rate as the population without diabetes (78). Moreover, according to the Juvenile Diabetes Research Foundation (JDFR), there were no COVID-19 deaths recorded among children with diabetes and the incidence of hospitalisation has been very low during the pandemic period (79). However, there are no available data regarding the incidence of cases with COVID-19 among patients with T2D. Curiously, this was completely the opposite of the situation among adults with diabetes, either T2D or T1D, who have been identified as one of the highest risk groups with an increased rate of hospitalisation (80). The risk of death due to COVID-19 in adults was about three times higher than the rest of the population as a whole (81). This could be related to the fact that children are less prone to serious COVID-19 infection as has been discussed earlier in this review.

Furthermore, it is well-known that diabetic ketoacidosis (DKA) rarely presents in new-onset cases with T2D, however, the COVID-19 pandemic has had a significant impact on increasing the risk of DKA among new-onset cases of T2D in adults (82, 83). The reason behind this might be that people are avoiding visiting medical centres and seeking medical advice (84). It is not clear whether COVID-19 has impacted the incidence of DKA among children with T2D and more scientific evidence is needed. DKA is an inflammatory condition associated with increased levels of several inflammatory factors including interleukin 6 (IL-6), interleukin-1β (IL-1β), and tumour necrosis factor (83). Therefore, this could have a worse impact by increasing the incidence of severe COVID-19 in patients with high risks, such as those who are obese and have a family history of T2D.

Even though the pathophysiological changes in diabetes patients with COVID-19 are not clear yet, this infection could lead to severe inflammatory cascade culminating in serious comorbidities (85). Moreover, it may trigger diabetes in many prediabetes cases or those at risk of developing diabetes, due to an increase in the levels of cytokines (86). This will be based on the fact that several viral infections increase insulin resistance, and as a result, the risk of developing diabetes (T1D and T2D) is very high (87). A good example of this is the hepatitis C virus, which has been found to be associated with a disturbance in β-cell function and inhibits the mechanism of glucose-stimulated insulin, *in vitro* (88). Furthermore, Yang et al. (89) have shown that the other coronaviruses, such as SARS-CoV, caused significant damage to different organs, including the lungs, kidneys, and the endocrine organs. This was directly related to a significant increase in the ACE2 expressions (the SARS coronavirus receptors) which explains the reason behind the development of acute diabetes in patients with SARS-CoV-2 who were previously healthy individuals (89). It has also been noticed that most of the cases recovered completely and that their diabetes reversed and only a few cases continued with chronic diabetes. Similarly, this was reported in some patients who had been affected by COVID-19 (85). It has been suggested that COVID-19 could trigger diabetes and thus indicates that there are significant complicated pathophysiological changes caused by COVID-19, concerning diabetes (85). There are reports that these cases were associated with poorer outcomes in comparison to patients with established T2D (9). For this purpose, there is currently a large international project known as CoviDIAB, organised by diabetes researchers worldwide (90). This could answer the most asked questions related to the risk of COVID-19 among children with diabetes, where most of the cases are mild.

It is not clear yet whether these risks could occur among children and adolescents with T2D or not. For this reason, vaccination against flu infections is recommended for people at risk such as people with obesity or with a strong family history of T2D and patients with diabetes during the current COVID-19 pandemic (91, 92). Although no scientific evidence has been provided yet, these groups of patients who are asymptomatic and have uncontrolled diabetes could be at risk of developing the symptoms of COVID-19. This could be triggered by increasing stress hormones and blood pressure, which could be developed due to the pandemic circumstances (93). Thus, psychological support for these patients could play a key role in protecting them. Patients with diabetes need to be reassured that their medical providers are accessible and available at any time either by phone or by email (94). Garge and his group (95) have found that during the COVID-19 pandemic, using telemedicine technologies to manage diabetes in new-onset T1D in children and adults is effective and feasible. Patients can share their data remotely with their physicians who can advise them and adjust insulin doses, accordingly, using emails, phones, and via video calls. Thus, identifying the feasibility of the virtual tools could be considered as one of the beneficial impacts of the pandemic as it will allow patients to seek medical advice at their convenience and is less stressful in terms of social distancing, travel, and missing school for some children (95, 96).

However, these facilities may not be available in some areas where the internet is not available. Therefore, other prevention approaches such as exercise and fasting for some patients could play a key role in reducing or eliminating hospitalisation and comorbidities. Advising patients to go outside for walks and practising light to moderate exercise would have a great impact (97). In addition, IF has been studied for years (98, 99). It has been indicated that the implementation of several fasting programmes into practice has the potential to improve the disease prognosis and can reverse the disease condition, particularly in patients with T2D and prediabetes (20, 100, 101). While this has been reported among adults with T2D (102), this approach has not been investigated widely among children and young adults. This article will discuss several types of fasting and it will introduce the importance of Ramadan fasting in more depth. Fasting in general is a cost-effective measure to treat and prevent several chronic illnesses such as diabetes. Authors of this article propose that applying this approach among children and young adults with T2D or at prediabetes stage, could be beneficial and a preventive and protective approach in terms of minimising the integrated risks of the two epidemics: diabetes and COVID-19. It is like any other approach that might work more for some people than others, but could save lives until accurate evidence/data regarding the effects of COVID-19 infections in these focused groups are identified and published. Furthermore, IF and changing life-style may prevent these young people from taking medications for the rest of their life.

## Intermittent Fasting

Intermittent fasting (IF) has been defined as periodic fasting where people are fasting and eating for certain hours during the day (103). Extensive research showed that IF is associated with numerous health benefits including extending life span, cognitive function, intellectual performance, and metabolic regulation among healthy adults and patients with different disorders (100, 104). Several studies suggested that IF could have the profound potential to be used as a preventive/therapeutic tool for chronic illnesses (100, 104). This is based on the fact that naturally and genetically, the human body system is programmed on periods of intermixture cycles: active and rest cycle, feast and famine cycle, where these intermittent periods are critical for the human physiology to be able to modulate all the metabolic and biological processes required (105). In addition, it has been proven that the other metabolic processes including the shift in energy sources during the fasting period are essential in providing the optimal energy for cellular functions and regeneration (106). The abolishing of these cycles, caused by eating frequently without proper physical activities as in a sedentary lifestyle, results in metabolic and biological deregulations and the development of different metabolic disorders, such as diabetes and obesity (100, 106). Various approaches of IF have been widely studied including alternate day fasting (ADF) and time-restricted feeding (TRF). Moreover, Ramadan fasting is also a kind of IF and it is often referred to as Ramadan intermittent fasting or Ramadan diurnal IF in the scientific literature (107, 108).

Alternate day fasting has been identified by fasting every other day and during the fasting day, the followed protocol is either to limit the food intake to only 25% of the daily food intake (500 calories/day) or to consume zero calories, while returning to the normal healthy diet during the eating day (101). On the other hand, TRF is characterised by the limitation of the daily consumed food over a specific period during the day with no calorie restriction and this time limit varies from 4 to 12 h (109). Considerable research attention has been paid to these kinds of fasting in humans and animals (98, 99, 110). It has been reported to be associated with a significant improvement in glucose homeostasis, blood pressure, decreased lipid biomarkers, lowering of inflammation, body weight reduction, insulin level, fasting blood glucose (FBG), and insulin sensitivities (20, 109, 111, 112). However, some scholars reported that ADF was associated with a remarkable rise in hunger during the fasting day making this approach unpleasant or inconvenient for a longer period (113). Another negative consequence of ADF is that people who are food lovers or heavy eaters did not lose much weight on this regime as they might be eating a large amount of food during the feasting day leading to hyperphagia (114). To prevent these drawbacks, this approach was replaced with TRF for some people.

Gow et al. (115) suggested that an intensive low-calorie diet could be used as a therapeutic tool for T2D among children and adolescents and it might be more efficient and able to cure the disease than standard medications. In their study, eight patients with T2D had a very low calorie/energy diet (VLED) at less than 3,360 kJ/day for 8 weeks followed by a hypo-caloric diet at about 6,300 kJ/day for 34 weeks. They reported that there were significant reductions in insulin level, weight, cholesterol level, HbA1c with a noticeable improvement in insulin sensitivity in all participants (115). Furthermore, three participants on insulin were able to stop their medication by week 8 and the other participants who were on metformin achieved T2D reversal by week 34 (115). However, in the opinion of this author, this extremely low-calorie diet pattern (including 3 to 4 meals of a low carb diet for 8 weeks, which is gradually restricted to one meal per day) might be considered as a tough lifestyle regime and it would probably not be followed by most of the patients of a younger age. This regime has also been evaluated among adults and up to now many studies have suggested that the main pathophysiological changes in diabetes; beta-cell failure and insulin sensitivity could be reversed by just following the VLED, consequently, disease remission was achieved in approximately half of the patients who adhered to this protocol (116–118).

Furthermore, an important study conducted in the UK by Lean et al. (119) reported that complete remission of T2D among young adults and adults was successfully achieved by following diet replacement over 12 months. This study conducted over 4 years was known as DiRECT (119). Thus, even though research among children and young adults with T2D is limited, specific diet regime such as VLED still has the potential to be used as a therapeutic approach for these patients who would like to avoid the use of medications and their adverse effects such as insulin. From this point of view, the diet pattern during RF could have the same potential positive impact, and research studies related to this are necessary as the diet approach could prevent disease complications, decrease health care costs, and positively influence the quality of patients' lives in the long term.

## Importance of Fasting In Reversing the Pathogenesis of Type 2 Diabetes and the Need for Studies In Children

Various theories have been reported to identify the reasons behind the disturbance in glucose homeostasis resulting in increased blood glucose, insulin level, and HbA1c, and consequently the development of diabetes (120). This includes environmental factors, a stressful life, sleep deprivation, and genetic factors (121, 122). However, it has been shown that this epidemic rise is strongly related to a substantial alteration in diet or lifestyle in general, where people tend to consume a great amount of processed foods, fast foods, and refined sugars (120). Dalgaard (120) has proposed that cells are protecting themselves from the high level of glucose by shutting off the glucose uptake to prevent any cellular damage that could take place due to auto-catalytic glycation. This was based on the theory of epigenetics by which the cells can regulate the expression and suppression of different genes and modify them according to the intracellular biological function, for instance when the cells are exposed to increased amounts of glucose (123). These genetic modifications are preventing the cells from taking more glucose from the blood, and this might be mediated by decreasing the expression of glucose transporter type 4 (GLUT4) and/or impairing the insulin receptors/insulin signalling pathway (124). Furthermore, several studies have shown that people with diabetes have certain epigenetic variations in comparison to healthy individuals (124, 125). This explains the improvements in insulin sensitivity that have been observed in some studies that are based on IF and calorie restriction approaches (115). Thus, changing diet by consuming low to no carbohydrates could reverse the condition and reactivate the genes and transcription factors that are necessary for glucose uptake. Therefore, in the case of insulin resistance and based on the above theory, T2D could be cured/reversed by just modulating diet such as by consuming fewer carbohydrates, and this has been already proved in some studies (115, 116, 126).

In recent decades, it has been shown that the incidence of insulin resistance has substantially increased among children (specifically at around 12 years old), adolescents, and young adults. This substantial rise was strongly associated with obesity and overweight epidemics among these age groups (127). Further, the negative effect of puberty on insulin sensitivity plays a role in the rapid progression of this disorder (128). This could be pertinent to hormonal and metabolic alterations among adolescents, where insulin sensitivity is significantly declined, and this alteration is automatically reversed later by the end of puberty (129). However, in children/adolescents who experienced obesity during their growing periods, this condition might remain and cause diabetes (129). Once β-cells fail to compensate for the insulin resistance, high-risk individuals progress gradually to pre-diabetes and eventually go on to develop diabetes (130). Moreover, it has been observed that the pathogenesis of T2D among adolescents and/or young adults (<20 years old) who are obese is somewhat similar to the pathological changes in adults, in terms of the reduction of β-cell function about a significant decline in insulin sensitivity (131). In addition, a failure in insulin secretion was observed even within overweight youth with a normal FBG and oral glucose tolerance test (127). Furthermore, Sjaarda et al. (132) found that in adolescents who had prediabetes, HbA1c between 5.7 and 6.5% had significant impairment in β-cell function.

Therefore, all these observations indicate that the administration of new dietary modification approaches such as IF among younger age groups could have a profound potential as a therapeutic and preventive regime. This could be an effective strategy for people who are at risk such as obese children/adolescents, in combination with physical activities and dietary interventions. Soliman et al. (133) have recently suggested the effects of IF in switching host metabolism. However, more scientific research is required in the near future in order to apply this in clinical practice. The standard treatment of these groups of the population starts with lifestyle alterations including nutritional advice and the encouragement of physical activities as it has been reported that loss of body weight by around 6 % has a significant impact on blood glucose control (134). A randomised controlled trial study conducted for around a year among obese 8–16 years old children found that an intensive family-based programme (nutrition, exercise, and changing behaviour) had a positive impact on insulin sensitivity and body composition indices such as weight, BMI, and body fat (134). Furthermore, Marcus et al. (135) have conducted the most popular study known as Treatment Options for T2D in Adolescents and Youth (TODAY) investigating the best therapeutic approach for those with T2D who are obese. They have noticed that apart from the medical treatment that has been prescribed, reduction in body weight is critical and associated with substantial effects on C-peptides, HbA1c, and lipid parameters (135). However, another study reported that dietary intervention by introducing low-calorie food was not effective among adolescents (136). It could be argued that this perhaps relates to the physiological and the biological variations among humans. Similarly, fasting programmes in general could be more beneficial for some people than others.

The therapeutic approach in early-onset T2D is based mainly on the hyperglycaemic state and the metabolic parameters, where patients are advised to start with metformin tablets either alone or in combination with insulin (127). Furthermore, the evidence displayed that different kinds of bariatric surgery such as laparoscopic adjustable gastric banding, Roux-en-Y gastric bypass, could be effective as a preventive and therapeutic approach for both early and late-onset T2D associated with severe obesity (137, 138). Bariatric surgery has profound useful impacts on regulating glucose homeostasis biomarkers in obese youth with and without diabetes, reducing coronary heart disease risk, and also giving complete remission to patients with T2D among adolescents compared to other medical treatments (139). The remission rate reached up to 90% in some surgery types, for instance, biliopancreatic-diversion (140). However, like any other surgery, it has some drawbacks or complications including hypoglycaemia, hernia, anastomotic leaks, ischemia, and pulmonary embolism (141). Thus, it will be more sensible to introduce safer programmes/approaches such as fasting to avoid all these risks and achieve the same results.

Another point to mention is that compared to T2D in adults, early-onset T2D has an aggressive nature and is associated with serious complications leading to an increase in rates of mortality and morbidity (142, 143). These include macrovascular complications, cardiovascular risk, and renal function disturbance; most of these complications are age-related meaning they tend to develop at an early age (143). This might be due to many factors such as psychological/social factors and the rate of response to the medications. In addition, it has been anticipated that this will get worse during the current pandemic circumstances due to the effects on the mental health of children and young adults (144). Therefore, new approaches including preventives and therapeutics are essential in order to reduce this epidemic and to provide a healthier life for this group of the population. RF is one of the most common types of IF that has been investigated among adults and mainly within Muslim communities, constituting around 1.9 billion worldwide (145). Early intervention in children and young people, through a combination of intermittent fasting, dietary guidance and physical activity may prevent or reverse diabetes and ensure that poor health does not persist into adulthood. RF where children fast for a month is a good opportunity that should not be missed. A “prevention is better than cure” approach is particularly important with childhood obesity reaching epidemic levels (146).

## Effects of Ramadan Fasting on Patients With Type 2 Diabetes Among Children and Young Adults

Most of the research that has been conducted pertaining to the effects of RF on glucose biomarkers in T2D patients was among adults and young adults (23, 147). The findings were controversial with wide variations in the study design and methods that were used to measure and assess the metabolic parameters (148). It has been reported that RF is safe and has a significant impact on weight reduction among adult patients with T2D, without a significant increase in the frequency of hypoglycaemia/hyperglycaemia when compared to controls (149, 150). Furthermore, RF is associated with a remarkable improvement in glucose lipid biomarkers including HbA1c, FBG, fractosamine, TG, and LDL (23, 151). All these findings indicate that RF could prevent/decrease cardiovascular disease risk in T2D patients. In addition, several studies reported that intensive education programmes before and during Ramadan have had a significant impact on improving and preventing the complications of diabetes such as hypoglycaemia, and this was in comparison to standard health care (152–154). Interestingly, it has been reported that the high similarity between RF and TRF makes it reasonable to translate the effect of TRF to RF (155).

To date, no attention has been paid to the effects of RF among children with T2D even though it is well known that children participate in RF. The impact of RF in glucose biomarkers among children and adolescents with T2D has not been examined yet. However, it has been reported that the effects of RF on children and adolescents have been examined mainly for T1D (156, 157). Evidence supported the fact that a majority, around 60 per cent of children and teenagers with T1D, can fast for more than half of the month of Ramadan and that they can fast safely in association with proper and focused education before Ramadan and close medical care during Ramadan, where patients are advised to break their fasting during hypo/hyperglycaemia (154, 158, 159). Similarly, and more recently Zabeen et al. (160) have concluded that children and adolescents with T1D and have uncontrolled blood glucose can observe Ramadan safely if they have been provided with close medical care. However, this kind of support may not be provided for Muslim children and adolescents who are interested to fast during Ramadan in Western countries. Misconceptions between paediatrics medical professionals and parents of fasted children in Michigan, US, has been reported (161). It has been found that no medical advice being provided for fasted children (161). In addition, differences in complication frequencies in people with T1D on an insulin pump compared to those on multiple-dose injections (MDI) were not be identified (159). Supporting this, Eid et al. (154) found that intensive/focused education programmes before/during Ramadan, are associated with a significant improvement in glucose homeostasis biomarkers (FBG and HbA1C).

On the other hand, other studies considered that children and young adults with diabetes as a high-risk group that should not fast during the month of Ramadan as it may increase the incidence of Diabetic ketoacidosis (DKA), dehydration, and hypoglycaemia among T1D in these age groups [reviewed by Beshyah et al. (157)]. Furthermore, most of the recent studies reported that RF was not associated with an increased risk of DKA (157). Therefore, even though the pathogenesis of T1D varies from T2D, the above evidence strongly support that RF could be very effective for some patients with T2D in children and adolescents. Examining how fasting could affect children's health is vital and more research is needed. However, based on the currently available literature among adults, it might be safer to implement fasting programmes among healthy young adults and patients with controlled diabetes under close observation either to support them to practice RF or apply some previously studied programmes of IF.

## Ramadan Fasting During Covid-19 and Its Impact on Children and Young Adults With Diabetes

Recently, several reviews have examined the impact of fasting (IF and RF) on healthy people and patients with chronic problems; considering the influence of the COVID-19 pandemic, they reported that RF is safe among healthy people and some people with controlled diabetes among adults (162, 163). In addition, several beneficial effects have been reported among healthy adolescents, for instance decreasing the incidence of obesity, preventing infections, and mental disorders (164). Furthermore, the importance of combination of RF, exercise and good nutrition was recommended to boost the immune system among Muslims societies during COVID-19 pandemic before Ramadan 2020 (165). No study so far has identified the impact of COVID-19 pandemic during RF on children and young adults with T2D. Regardless, it can be argued that due to the presence of lockdown measures such as school closures during the previous Ramadan, these children may have fasted more safely where they had more rest and increase in sleeping hours in the morning. This could outweigh the health outcomes of RF and improve the blood glucose parameters and the disease prognosis in general. However, they might struggle to obtain the appropriate medical support needed. Therefore, more scientific studies are required to identify how these patients manage their fasting during Ramadan and whether they have been provided with medical advice or not.

Despite the fact that children with T2D have not been identified as a high-risk group for COVID-19, precaution is essential among these patients, and focus group education would be beneficial before the month of Ramadan. For example, advice for patients on how to keep hydrated, consuming healthy food, adjusting medications and physical activities during Ramadan. This will avoid any risk of hypoglycaemia that may occur, which has been reported to be related to the age group among children (166, 167). Moreover, the physiological mechanisms of maintaining the normal blood glucose level during the fasting period between children and adults are slightly different (166, 168). However, this case could be only among younger children, less than 10 years old who have a rapid reduction in blood glucose level and increase in the ketones levels compared to older children during fasting (168). Children who are expected to fast during Ramadan are usually at the age of puberty, between 12 and 15 years old. Recently Diabetes and Ramadan (DAR) International Alliance (https://daralliance.me/) community has published an updated practical guideline to help the medical professionals to support patients with diabetes who are interested to fast during Ramadan (169). Unfortunately, this does not cover guidelines for children and there is, as yet, no guideline that proposes IF as a preventive and protective strategy against diabetes. Furthermore, the guideline is not implemented in all medical practices in western countries, and the most common practice is to discourage these people from fasting, but not all patients are following this advice. Thus, there is an urgent need for supporting these patients medically as this will help to understand and classify who could have benefits or drawbacks of fasting, particularly in children and young adults with T2D at their early stage of the disease.

## Conclusion

In general, it is evident that there is great reassurance about the impact of COVID-19 infections on children and young adults. Most of the cases have milder symptoms and have an excellent prognosis. Even patients with diabetes, have the same risk of infection as those without diabetes. However, according to the pathophysiological changes from diabetes, some patients with T2D could be at risk of comorbidity in the case of any infection including SARS-CoV-2. Moreover, the data available are very limited in terms of new-onset diabetes in relation to the COVID-19 infection and the risks of DKA among children with T2D. The impact of the pandemic circumstances on the rates of identifying new cases among people at risk, such as those who have prediabetes, has not been well investigated. The current data highlight the importance of introducing and implementing preventive and protective tools during the current period of uncertainty. This could include encouraging physical exercise, healthy diet, and practising IF and RIF by some patients who have well-controlled diabetes or at prediabetes stage. These kinds of preventive and protective approaches will be paramount in improving public health, and significantly decrease the burden on health care providers. Unfortunately, there is a lack of studies on IF in children with T2D even though it is known that many of these patients fast during the month of Ramadan. There is, therefore, a definite need for patients who are willing to observe the month of Ramadan so they can achieve the benefit of fasting safely under medical supervision and potentially reverse their diabetes. In addition, more studies are required in order to obtain a clearer understanding of the biological effects of COVID-19 among children and young adults with T2D. Greater efforts are needed to ensure the effectiveness of fasting in patients with T2D among children and young adults and how this may help people who are at risk of developing diabetes during stressful situations such as pandemics. Another important practical implication is that even though conducting experimental studies is a great research challenge during the pandemic restrictions, using virtual tools such as survey-based studies or video interview-based studies, may have a great influence on clinical care, patient support, and in developing novel and effective guidelines. These kinds of studies could be conducted in all paediatric centres among T2D cases in children and young adults. Furthermore, they could also explore the percentage of patients who developed diabetes due to COVID-19, the risk of DKA and comorbidity in patients with established diabetes and confirmed COVID-19.

## Author Contributions

HE suggested the idea and the importance of the whole review and carried out most of the work, including searching, structuring, writing up and editing the review article. She contacted all the authors and discussed the importance of all the suggested points with them. MF provided detailed review of the article and helping in the editing process of the final drafts. He has also provided several recent studies/information to support some insights in the review. SU provided detailed review to this piece of work and has suggested several ideas in reorganising the article. SG provided a comprehensive overview of this work and suggested some ideas to make it more sensible and easier to read by many people from different fields. JG provided a deep review of the article and updated information to support some points. PH is a Ph.D. supervisor of HE and provided many updated resources to enhance the quality of this work. In addition, he has helped in reviewing and editing of the final drafts. A-BA-M is a Ph.D. supervisor of HE and is the corresponding author responsible of the publication process. He contributed in the restructuring, editing process, and reviewing of the article. All authors contributed to the article and approved the submitted version.

## Conflict of Interest

The authors declare that the research was conducted in the absence of any commercial or financial relationships that could be construed as a potential conflict of interest.

## Publisher's Note

All claims expressed in this article are solely those of the authors and do not necessarily represent those of their affiliated organizations, or those of the publisher, the editors and the reviewers. Any product that may be evaluated in this article, or claim that may be made by its manufacturer, is not guaranteed or endorsed by the publisher.
